# Combining Survey and Social Media Data: Respondents' Opinions on COVID-19 Measures and Their Willingness to Provide Their Social Media Account Information

**DOI:** 10.3389/fsoc.2022.885784

**Published:** 2022-07-06

**Authors:** Markus Hadler, Beate Klösch, Markus Reiter-Haas, Elisabeth Lex

**Affiliations:** ^1^Department of Sociology, University of Graz, Graz, Austria; ^2^Department of Sociology, Macquarie University, Sydney, NSW, Australia; ^3^Institute of Interactive Systems and Data Science, Graz University of Technology, Graz, Austria

**Keywords:** survey, social media, Facebook, Twitter, polarization, COVID-19, sentiment analysis, qualitative content analysis

## Abstract

Research on combining social survey responses and social media posts has shown that the willingness to share social media accounts in surveys depends on the mode of the survey and certain socio-demographics of the respondents. We add new insights to this research by demonstrating that the willingness to share their Facebook and Twitter accounts also depends on the respondents' opinions on specific topics. Furthermore, we extend previous research by actually accessing their social media accounts and checking whether survey responses and tweets are coherent. Our analyses indicate that survey respondents who are willing to share their social media accounts hold more positive attitudes toward COVID-19 measures. The same pattern holds true when comparing their sentiments to a larger Twitter collection. Our results highlight another source of sampling bias when combining survey and social media data: a bias due to specific views, which might be related to social desirability.

## Introduction

Combining survey and social media data has become more common over the last few years (Hill et al., 2019; Stier et al., 2019). Researchers have used this approach to study substantive questions such as political preferences and filter bubbles (Eady et al., 2019; Wolfowicz et al., 2021), and to discuss ethical challenges (Breuer et al., 2020, Sloan et al., 2020). This approach has also been used to address methodological issues such as using tweets to validate survey responses (Henderson et al., 2021) and researching possible biases in the willingness to share data (see Al Baghal et al., 2019; Mneimneh et al., 2021). Our paper focuses on the latter, thus expanding the research on biases in the willingness to share data. In other words, our research focuses on the differences between survey respondents who share their social media accounts and those who do not by considering attitudes and by accessing the respondents' social media accounts.

Previous research has shown that the willingness of survey respondents to provide additional data is limited. Revilla et al. (2019) provide an overview of various studies that consider the readiness of survey respondents to use additional tools such as geolocating, GPS tracking, and visual data capturing. The reported values varied widely. For example, the readiness to allow GPS tracking was in the 30% range, yet was around only 11% for the readiness to allow the respondent's child to wear a wristband that sends information to an online site. Studies considering the consent to share social media accounts report a willingness of between 24 and 45% (Al Baghal et al., 2019; Mneimneh et al., 2021).

Given that only a limited number of survey respondents grant access to their social media accounts, combined data sets capture only a fraction of the data from the total number of survey respondents who actually use social media. Thus, using such samples to draw conclusions regarding an entire population is problematic, at the very least (Sen et al., 2021). If there is any systematic deviation, in the sense that certain groups are not willing to share their data, the linked dataset is biased. Al Baghal et al. (2019) show that the rate of agreement varies with the mode of the survey—with higher consent rates in face-to-face surveys—and with differences regarding gender and age, although those effects are inconsistent. Mneimneh et al. (2021) demonstrate that self-reported Twitter usage patterns can be important predictors of the willingness to share accounts. Henderson et al. (2021) were aware that consent to provide access is non-random. Therefore, they compared sample characteristics in their research, which indicates minor differences in terms of age, gender, education, race, and income.

The studies cited in this previous paragraph provide critical insights into this topic. Yet, our paper addresses novel aspects. While Al Baghal et al. (2019) and Mneimneh et al. (2021) base their analysis on the reported willingness to provide access, we also access the social media accounts of our respondents and thus can check whether respondents actually provide valid account names. Henderson et al. (2021) compared verified Twitter users to all Twitter users in their survey, but did not consider non-users or users whose accounts were not verified. We considered all these groups and conducted multivariate analyses testing the effects of various socio-demographics and attitudes on the respondents' willingness to share their account information. Specifically, we considered the effects of the respondents' opinions on COVID-19 measures.

Opinions on COVID-19 seem to be particularly suited for our purpose, as the pandemic gave rise to various conspiracy theories and associated skepticism toward science and scientific advice (Chayinska et al., 2021; Priniski and Holyoak, 2022). Our expectation was that respondents who oppose the COVID-19 measures are less likely to grant access to their social media accounts. One possible explanation^1^ to consider is “social desirability.” The social desirability bias assumes that respondents would like to be seen in a favorable light by the researcher and are thus likelier to underreport socially undesirable views and actions (Phillips and Clancy, 1972; Krumpal, 2013; Henderson et al., 2021). Social desirability effects are weak in anonymous settings, such as online surveys. They are more significant in face-to-face interviews and when the respondent is known to the researcher. Providing access to one's social media account can remove the anonymity of an online survey, especially when the social media account reflects the real name of a respondent or includes additional information that allows identification of the account holder. Given that we inform our respondents that we collect the social media information for a scientific purpose and that previous research indicates science skepticism among COVID-19 deniers, we expected, as stated above, a lower consent rate among respondents who oppose the COVID-19 measures.

In sum, these considerations lead to the following main research hypotheses: (a) The willingness of survey respondents to share social media accounts depends on their attitudes toward a specific topic, in our case, COVID-19 measures. We expect (b) a bias due to socially desirable responses, e.g., a higher willingness to share data among respondents who are in favor of COVID-19 measures. Finally, we will also investigate whether there is a difference between respondents who consent to share their data and respondents whose accounts can be accessed successfully.

The following section explains how the data was collected and used for our study. The data consists of an online survey of the adult population of Austria, Germany, and the German-speaking parts of Switzerland; the tweets of our survey respondents; and all tweets that match predefined search parameters related to COVID-19 during the survey period. The results section starts with a report on the different social media usage patterns of our survey respondents and their willingness to share Facebook and Twitter accounts. Afterwards, we compare the attitudes expressed in our survey to related tweets that were crawled during the survey period. Overall, our findings confirm our expectation that respondents who share their social media data hold more positive opinions toward the COVID-19 measures, and that merged data sets can be biased with regard to specific opinions.

## Methods

Our analyses are based on a public opinion survey, the tweets of survey respondents who provided their account information, and Twitter data during the same period of time. The cross-sectional survey was fielded online in the summer of 2020 in Austria, Germany, and the German-speaking parts of Switzerland. A total of 2,560 individuals participated. The individuals were selected based on representative quotas reflecting the official distribution of gender, age, and federal state/canton in the three countries. Having met these quotas, it can be assumed that the sample resembles the characteristics of the total population. However, it cannot be considered a random sample. Therefore, we do not draw any conclusions regarding the general population and focus only on the biases within our sample.

Of the survey respondents, 67% were from Germany, 22% from Austria, and the remaining 11% from Switzerland. Austrians were oversampled due to a specific interest in regional differences by some members of our research team. The survey included questions on attitudes toward the COVID-19 crisis, the use of various social media platforms, and socio-demographic information. Respondents were also asked if they were willing to provide the name of their Facebook and Twitter handles and were subsequently asked for them.

Linking survey data with Twitter data requires specific ethical considerations (Sloan et al., 2020). Our respondents were informed about the content of this research, that their participation is voluntary, and that all information will remain confidential. Hence, the stored data does not include any information that would allow others to identify a specific person, including Facebook and Twitter handles or any tweets. The survey data, including more details on the fieldwork, are available publicly *via* Hadler et al. (2021)^2^.

The data for the dependent variable—the willingness to provide account information—was first derived from the responses to the public survey. This data includes the following groups for both Facebook and Twitter, respectively: (a) respondents without an account, (b) account holders who are not willing to provide their account information, and (c) holders who provided their account information. For Twitter users among our survey respondents, we were able to identify another group: (d) respondents whose Twitter accounts were accessed successfully. As for Facebook, we were not able to access these accounts as we do not possess the required licenses mandated by Facebook's terms and conditions.

The independent variables in this study (see Table 1) are the socio-demographics of age, gender, and education. We included these basic socio-demographic variables because, in related research, they were considered to have had some effects (see Al Baghal et al., 2019; Henderson et al., 2021; Mneimneh et al., 2021). In addition, we captured attitudes toward three COVID-19 measures that were the subject of intense political and social debate prior to and during our fieldwork: (1) “Once there is a vaccine against the coronavirus, there should be a mandatory vaccination for all;” (2) “To contain the spread of the coronavirus, contact tracing data (e.g., *via* apps) should be collected;” and (3) “I only wear a face mask when it is required by the government, and not voluntarily.” Responses were measured on a five-point scale, where 1 = absolutely disagree, and 5 = absolutely agree. Since the third item regarding the wearing of face masks was formulated inversely, we recoded it prior to the analysis. The three variables all correlate significantly with each other and have a Cronbach's alpha reliability score of α = 0.634 when combined to a single scale. Given the moderate Cronbach's α, we also include the three items separately in our regressions and report these results as noted in our tables.

**Table 1:** Characteristics of the survey sample.

**Variables**	**Mean (SD) or %**
Social media usage: see Table 2	
Opinions toward COVID-19 measures (1 = disagree and 5 = agree):	
Once there is a vaccine against the coronavirus, there should be mandatory vaccination for all.	3.19 (1.52)
To contain the spread of the coronavirus, contact tracing data (e.g., *via* apps) should be collected.	3.10 (1.39)
I only wear a face mask when it is required by the government, and not voluntarily.	2.99 (1.51)
Index	3.10 (1.12)
**Socio-demographic variables**	
Female	50.4%
Age	44.34 (13.80)
**Education**	
Compulsory school	35.2%
Vocational training	11.6%
High school degree	23.9%
University degree	29.3%

*n = 2,560; online survey conducted in Austria, Germany, and Switzerland in 2020. See methods section for details*.

We were able to collect 221 tweets from the Twitter accounts provided by our survey respondents. We conducted a qualitative content analysis of these tweets, as they are a special type of text material that is limited to 280 characters and often contains answers to previous tweets, links, images, and more. Furthermore, we wanted to capture explicit opinions about the COVID-19 measures and the pandemic and not rely on automated or standardized methods. Therefore, the tweets were coded inductively using the qualitative content analysis approach, and agreement with the COVID-19 measures was assigned manually by two researchers independently using an ordinal 5-point scale, as in the survey. Finally, these scores were compared in terms of congruence with the survey data of the respective respondents.

Our third source is a collection of tweets posted on COVID-19-related topics during the survey period. We used the keyword and account list from Chen et al. (2020) to collect German tweets from the full-archive search API (Application Programming Interface) using Academic Research access *via* twarc2. We filtered the tweets according to three predefined word stems that resemble the three prevention measures. Specifically, we used the German word stem “impf” for vaccination (*n* = 12,336 tweets), “trac” for contact tracing (*n* = 1,391), and “mask” for mask wearing (*n* = 35,044). As this selection includes all relevant tweets during this period, we use the commonly used data-sciences term “collection” for this source. Subsequently, we conducted a sentiment analysis using the Python library TextBlob with the German language extension, which includes a polarity lexicon for sentiment extraction. The extracted sentiments range on a scale of −1 for negative sentiment to +1 for positive sentiment. Similar to Al Baghal et al. (2021), we averaged the sentiment per Twitter account and excluded accounts that did not express any relevant sentiment or did not contain sufficient information for extracting a sentiment.

## Results

### Social Media Usage of Our Survey Respondents and Access to Their Social Media Accounts

Table 2 provides, first, a descriptive overview of the social media usage of our survey respondents and their willingness to provide their account information. These results are based on a set of questions that were asked toward the end of our survey: (1) “Do you have a private Facebook account? (yes/no);” (2) “How would you describe the way you use Facebook? (actively posting vs. more passively reading);” (3) “We would like to find out who is using Facebook and for which purposes. If you provide us access to your account, we assure to keep your personal information confidential (access granted vs. no access);” and (4) for those who granted access, “What is your username?” The same set of questions was also used for Twitter. Second, Table 2 depicts the number of accounts we accessed successfully on Twitter.

**Table 2:** Usage of different platforms and the willingness to provide access (survey data).

	**Facebook**		**Twitter**	
Account holders (% of all respondents)	1,774	69.3%	404	15.8%
Active users (% of holders)	700	39.5%	141	34.9%
Account provided (% of holders)	617	34.8%	119	29.5%
Successfully accessed (% of holders)	N.A.^*^		79	19.6%
*n*	2,560		2,560	

* *Account access restricted by Facebook's terms and conditions*.

The data shown in Table 2 confirms the already known difference between the usage of Facebook and Twitter in German-speaking countries in the sense that the former is used far more often by our respondents (69 vs. 16%). However, the proportion of account holders who consider themselves active users and of survey respondents who are willing to provide their account information are much more similar for both platforms. Around 40% of the Facebook account holders consider themselves active users, while around 35% of the Twitter account holders consider themselves active users. As for the willingness to provide their account information, 35% of the Facebook account holders provided their information, and around 30% of the Twitter account holders provided theirs. As for actual access to their social media accounts, we did not access the Facebook accounts due to the specific terms and conditions of the platform. As for Twitter, we were able to access the accounts of 79 respondents (20% of the account holders among our respondents). Forty respondents provided an incorrect Twitter name or a protected account.

### Attitudes Toward the COVID-19 Measures Across Different Social Media Platforms and Users

One of the main goals of our paper is to analyze the association between attitudes toward the COVID-19 measures and the willingness to share one's social media information in a public opinion survey. Table 3 provides several statistics for all users of a platform compared to our respondents and users who granted access. Furthermore, we provide additional details for Twitter users among our survey respondents, as we were able to differentiate between accounts that we accessed successfully and accounts that we could not access. The bottom part of Table 3 presents statistics based on tweets: first, the sentiments shown in the tweets of our survey respondents and second, the sentiments shown in the overall collection of tweets during the survey period.

**Table 3:** Different usage groups and their opinions on COVID-19 measures.

**Dataset and variables**	**Statistics**	**Mean**	**Min**	**Max**	**1. Quartile**	**2. Quartile—Median**	**3. Quartile**	***n***
All survey respondents	Vacc.	3.19	1	5	2	4	5	2,497
	Mask	2.99	1	5	2	3	4	2,523
	CT	3.10	1	5	2	3	4	2,502
	Index	3.09	1	5	2.33	3.33	4	2,541
Survey respondents who have a Facebook account	Vacc.	3.12	1	5	2	3	5	1,732
	Mask	2.95	1	5	2	3	4	1,752
	CT	3.10	1	5	2	3	4	1,735
	Index	3.06	1	5	2.33	3.33	4	1,761
Survey respondents who provided us with their Facebook account name	Vacc.	3.27	1	5	2	4	5	603
	Mask	2.95	1	5	2	3	4	609
	CT	3.34	1	5	2	4	4	608
	Index	3.18	1	5	2.33	3.33	4	612
Survey respondents who have a Twitter account	Vacc.	3.28	1	5	2	4	5	396
	Mask	3.27	1	5	2	4	5	401
	CT	3.28	1	5	2	4	4	399
	Index	3.27	1	5	2.33	3.33	4	403
Survey respondents whose Twitter accounts were accessible	Vacc.	3.24	1	5	2	4	4	78
	Mask	3.38	1	5	2	4	4	79
	CT	3.56	1	5	3	4	4	79
	Index	3.40	1	4.67	2.67	3.67	4	79
Survey respondents who tweeted about COVID-19	Vacc.	3.53	1	5	2	4	5	19
	Mask	3.60	1	5	3	4	4.75	20
	CT	3.75	1	5	4	4	4	20
	Index	3.63	2	4.67	3.08	3.83	4.33	20
Twitter accounts that express sentiment regarding COVID-19 measures in the survey time period	Vacc.	0.21	−1	1	−0.19	0.23	0.7	4,465 (8,344 tweets)
	Mask	0.02	−1	1	−0.17	−0.03	0.25	11,537 (26,029 tweets)
	CT	0.16	−1	1	−0.18	0.23	0.44	673 (865 tweets)
	Index	0.08	−1	1	−0.15	0.06	0.33	14,752 (36,769 tweets)
Twitter accounts of survey respondents that express any sentiment regarding COVID-19	Index	0.16	−0.08	0.5	0.02	0.15	0.28	19 (220 tweets)

*Results from the survey data are based on the questions: “Once there is a vaccine against the coronavirus, there should be mandatory vaccination for all.” “To contain the spread of the coronavirus, contact tracing data (e.g., via apps) should be collected.” “I only wear a face mask when it is required by the government, and not voluntarily.” With 1 = disagree and 5 = agree. Additionally, we calculated all measures by excluding the value “3” which equals “neither/nor” and might match the neutral sentiment in the sentiment analysis of Twitter. The substantive findings remain the same*.

*Results from Twitter are based on sentiment on the three prevention measures during the survey period. The sentiments are measured as per tweet in a range from −1 for the maximum negative sentiment to +1 for the maximum positive sentiment. The numbers presented in Table 3 are based on the average of all sentiments of an account. Accounts that do not express any sentiment and tweets with a neutral sentiment are excluded*.

Before we discuss the survey results and social media content, we also have to ensure that there is a match between an individual response in the survey and the respondent's postings on social media. This initial step is necessary to ensure that our comparison of survey responses and Twitter sentiments is valid. Of the 79 Twitter accounts that were successfully accessed, 20 accounts (i.e., survey participants) posted about the COVID-19 pandemic. Overall, a total of 221 tweets from these 20 accounts were analyzed using qualitative content analysis. All tweets were original postings (i.e., no re-tweets). The manual classification of the tweets by two independent researchers regarding the user's opinion toward the pandemic and accompanying measures shows a binary inter-annotator agreement of α = 0.7. The assessment of the coherence between (a) the survey answers of our respondents, (b) their tweets on the COVID-19 measures, and (c) their overall COVID-19 Twitter sentiments shows a match in all but eight total cases (out of 42 pairwise comparisons^3^)—that is, a match of 81%. This agreement indicates a relatively high level of congruence regarding the opinions toward the pandemic and the related measures between the survey data and the Twitter data, which lends support to our following comparison of survey results and sentiment analysis.

Table 3 shows that the Twitter account holders among our survey respondents are generally more in favor of the COVID-19 measures than the overall sample, whereas Facebook account holders express more of an average sentiment. The mean value across the three COVID-19 measures—vaccination, mask wearing, and contact tracing (on a scale of 1–5, 1 = absolutely disagree)—is 3.09 for the overall survey respondents, 3.27 for Twitter account holders, and 3.06 for Facebook account holders. A positive bias is visible for respondents who are willing to share their Facebook account (mean = 3.18), respondents whose Twitter accounts were actually accessed (mean = 3.40), and even more for respondents who actually posted on Twitter on this topic (mean value = 3.63).

Alongside the survey data, we also analyzed Twitter data. The sentiment analysis considered all German tweets published during the same time period as our survey and that included either positive or negative sentiments regarding the three COVID-19 measures. To deal with the fact that a single account can post multiple tweets and thus lead to an imbalance in numbers (Al Baghal et al., 2021), we based the statistics in Table 3 on the average value for each account. The results show that the overall sentiment regarding the three COVID-19 measures is 0.08. The actual tweets of our 20 survey respondents who posted on COVID-19-related matters indicate a mean value of 0.16, which is larger than the average within the Twitter collection. Hence, we observe the same bias in the tweets as in the survey data—the tweets of respondents who shared their account information are more positive toward the COVID-19 measures than those of the larger Twitter collection.

### Multivariate Analyses of Factors Influencing the Willingness to Share Social Media Content

So far, we have presented various descriptive analyses of attitudes toward COVID-19 measures and the willingness to share social media content. Table 4 presents the results of three multinomial regression models that estimate the effect of these COVID-19-related attitudes on the willingness to share account information, controlling for a few socio-demographic variables. The first two regression models use “respondents who are willing to provide their account information” as the reference group being compared to respondents who do not have an account as well as those who did not provide their account information in the survey. The third regression model allows another differentiation for Twitter, as it uses “respondents whose Twitter accounts were actually accessed” as the reference group being compared to “respondents without an account,” “respondents who have an account but did not grant access,” and “respondents whose accounts were not accessible” (due to incorrect account names or protected accounts).

**Table 4:** Social media usage and the willingness to provide account information (Multinomial regression).

	**Facebook**		**Twitter**		**Twitter**		
	**(ref: account provided)**		**(ref: account provided)**		**(ref: account accessible)**		
	***B*** **-values (** ***p*** **-values)**		***B*** **-values (** ***p*** **-values)**		***B*** **-values (** ***p*** **-values)**		
	**No Account**	**Account, but not provided**	**No Account**	**Account, but not provided**	**No Account**	**Account, but not provided**	**Account provided but not accessible**
Intercept	−0.756 (0.016)	0.647 (0.023)	1.389 (0.009)	1.445 (0.019)	1.337 (0.041)	1.397 (0.052)	−2.117 (0.055)
Pro COVID-measures	−0.069 (0.175)	−0.162 (0.001)	−0.279 (0.003)	−0.105 (0.327)	−0.343 (0.003)	−0.170 (0.184)	−0.185 (0.328)
Female	−0.162 (0.144)	0.141 (0.165)	0.726 (0.000)	−0.065 (0.776)	0.939 (0.000)	0.147 (0.590)	0.618 (0.125)
Age	0.033 (0.000)	0.008 (0.056)	0.029 (0.000)	0.000 (0.983)	0.040 (0.000)	0.011 (0.279)	0.032 (0.031)
Compulsory school	−0.192 (0.165)	−0.205 (0.107)	0.206 (0.402)	−0.232 (0.413)	0.088 (0.768)	−0.348 (0.292)	−0.350 (0.486)
Vocational training	−0.234 (0.226)	−0.188 (0.287)	0.191 (0.592)	−0.453 (0.288)	0.123 (0.781)	−0.521 (0.298)	−0.200 (0.783)
High school degree	0.342 (0.028)	0.152 (0.288)	−0.029 (0.906)	−0.155 (0.581)	−0.054 (0.856)	−0.180 (0.583)	−0.085 (0.865)
University degree	Ref.		Ref.		Ref.		
Cox-Snell	0.042		0.048		0.051		
Nagelkerke	0.047		0.074		0.075		
*X^2^* (*df*)	108.039^***^ (12)		125.215^***^ (12)		132.230^***^ (18)		
*n*	2,529		2,529		2,529		

*Pro-COVID-19 measures (low value = disagreement); age in years; education (reference category = University degree)*.

*We have also taken into account the opinions on the three respective COVID-19 measures separately. This analysis shows that the item on mask wearing is only significant regarding the difference between the reference group and respondents who do not have an account. As for Twitter, the item on contact tracing becomes significant and indicates that respondents, who oppose contact tracing, are also less likely to share their information*.

*We also considered excluding the middle category (3 = “neither nor,” on a scale from 1 to 5) for the variables regarding the COVID-19 measures in the survey data, as we did with tweets with a neutral sentiment. The results are very similar throughout*.

*** *p < 0.001*.

For Facebook, respondents without an account are older than the reference group, but, otherwise, do not differ significantly from the reference group in terms of gender, education, and attitudes toward COVID-19 measures. For the respondents who do have a Facebook account but are not willing to share their social media information, the regression identifies a significant effect with COVID-19 attitudes. Facebook account holders who oppose the COVID-19 measures are less willing to provide their account information than Facebook users who support the COVID-19 measures. Socio-demographics, on the other hand, do not have significant effects for this group.

As for Twitter, the results indicate that respondents without an account are older and more often female when compared to the reference group, and are also more often against the COVID-19 measures. Regression model 2 does not indicate any significant differences between respondents who are willing to provide their Twitter accounts and respondents who are not willing to provide their accounts in terms of the variables considered when using the COVID-19 index. Comparing the groups of Twitter account holders whose accounts were accessible and those who (accidentally) provided an incorrect account name shows that older respondents more often reported an incorrect or protected account. Furthermore, when including the three COVID-19 measures separately, the effect of contact tracing becomes significant and indicates that respondents, who oppose contact tracing, are also less likely to share their information Hence, this specific aspect is also associated with the willingness to grant access.

Finally, the explained variances remain low in our models, with Nagelkerke values between 0.047 and 0.075. Given that a sampling error can be divided into a random part and a systematic bias (Sen et al., 2021; see also their supplemental material), a perfect random selection of respondents would be reflected in the absence of any systematic bias (i.e., showing no significant effects of any independent variables and a very low explained variance). Several of our variables are significant and thus indicate a systematic bias between account holders who share their information, those who do not share their information, and, to a certain extent, those who provide incorrect Twitter handles.

## Discussion

Our paper set out to investigate the association between attitudes toward the contentious topic of COVID-19 measures and the willingness of survey respondents to provide access to their social media accounts. The overall willingness to provide account information was around 30%, which is more or less in line with the numbers reported in previous studies (Al Baghal et al., 2019; Mneimneh et al., 2021). The overall willingness to provide information did not differ across the socio-demographic variables.

As for attitudes toward the COVID-19 measures, we found that respondents who oppose the measures are less willing to provide their Facebook account information. As for Twitter, the survey showed that Twitter users are generally more in favor of the COVID-19 measures than the other respondents. However, the same negative (albeit not significant) effect of less willingness to provide account information was also visible and in line with the Facebook findings. Furthermore, a separate analysis of the three measures showed that the item on contact tracing would be significant for Twitter as well. In sum, the contentious topic of COVID-19 measures is associated with the willingness to provide social media account details. These findings support our research hypotheses (a) and (b), that opinions on a specific topic are another source of possible biases when asking survey respondents for consent to share their social media accounts. Furthermore, our analysis also shows that younger Twitter account holders reported incorrect account information or a private account less often than male respondents. In line with our research hypothesis (c), we also see a bias between the sample of “consent given” and its subsample “account accessed successfully.” Our results thus add three additional aspects to the results discussed in previous papers (see, for example, Al Baghal et al., 2019; Henderson et al., 2021; Mneimneh et al., 2021).

A tentative explanation for this bias is social desirability (Phillips and Clancy, 1972; Krumpal, 2013; Henderson et al., 2021). Social desirability plays a role when survey respondents are no longer anonymous, which is the case, for example, whenever their social media handle allows them to be identified. In line with our research hypothesis (b), respondents who are in favor of the COVID-19 measures and thus aligned with scientific views are more willing to share their social media accounts with scientists. This explanation, however, remains tentative, as we did not include any questions on the reasons why respondents are willing to share their data. Yet, in a related paper (Klösch et al., 2022), we found that attitudes toward the environment are also associated with the willingness of our respondents to share data. Thus, it seems to be the case that attitudinal dimensions are related to the survey respondents' willingness to share their social media accounts.

Our research has other limitations. In terms of total survey error and its version for online data (Sen et al., 2021), our Twitter collection is limited to tweets that used the three predefined terms and our online access panel to registered respondents. Thus, our results should not be used to draw conclusions regarding the overall population. The matching of survey responses and tweets at the individual level was based only on a limited number of respondents, who, in addition, expressed rather positive views on the COVID-19 measures. This limitation is caused by, first, a shrinking sample size, as not all survey respondents use social media; second, the fact that not all respondents shared their account information; and third, the fact that not all accounts can be accessed. Furthermore, opinions can only be compared if they are expressed. Hence, we can only grasp the views of social media users if they expressed their opinion on COVID-19 measures—a group that constituted 20 account holders in our case.

In sum, we were able to demonstrate that attitudes toward a specific topic are associated with the survey respondents' willingness to grant access to their social media accounts, which adds another dimension to existing research on this topic. We were also able to show that the responses in the survey and the tweets are mostly coherent. Given the limitations in terms of sample size and Facebook account access, future research should revisit this topic using additional social media platforms and larger collections of actual users.

## Data Availability Statement

The datasets presented in this study can be found in online repositories. The names of the repository/repositories and accession number(s) can be found below: “(Hadler et al., 2021), ‘Polarization in public opinion: Combining social surveys and big data analyses of Twitter (SUF Edition)’, https://doi.org/10.11587/OVHKTR.”

## Ethics Statement

Ethical review and approval was not required for the study on human participants in accordance with the local legislation and institutional requirements. The patients/participants provided their written informed consent to participate in this study.

## Author Contributions

All authors listed have made a substantial, direct, and intellectual contribution to the work and approved it for publication.

## Funding

This work was supported by TU Graz Open Access Publishing Fund.

## Conflict of Interest

The authors declare that the research was conducted in the absence of any commercial or financial relationships that could be construed as a potential conflict of interest.

## Publisher's Note

All claims expressed in this article are solely those of the authors and do not necessarily represent those of their affiliated organizations, or those of the publisher, the editors and the reviewers. Any product that may be evaluated in this article, or claim that may be made by its manufacturer, is not guaranteed or endorsed by the publisher.

## Footnotes

^1^Other possible explanations are incentives, privacy concerns, social trust, and trust in institutions, given that previous research on this subject has established links between COVID-19 and these aspects (see, e.g., Bian et al., 2020; Hafner-Fink and Uhan, 2020; Kreuter et al., 2020).

^2^ https://doi.org/10.11587/OVHKTR

^3^Forty-two comparisons reflect the total number of possible pairwise comparisons, i.e., a respondent had valid answers in at least two variables.
